# Mouthwash Formulation Co-Delivering Quercetin and Mint Oil in Liposomes Improved with Glycol and Ethanol and Tailored for Protecting and Tackling Oral Cavity

**DOI:** 10.3390/antiox11020367

**Published:** 2022-02-11

**Authors:** Ines Castangia, Maria Manconi, Mohamad Allaw, Matteo Perra, Germano Orrù, Sara Fais, Alessandra Scano, Elvira Escribano-Ferrer, Mansureh Ghavam, Maryam Rezvani, Maria Letizia Manca

**Affiliations:** 1 Department of Scienze della Vita e dell’Ambiente, Drug Science Division, University of Cagliari, 09124 Cagliari, Italy; inescastangia@tiscali.it (I.C.); allaw.mohamad.22@gmail.com (M.A.); Matteo.perra@unica.it (M.P.); maryrezvani.unica@gmail.com (M.R.); mlmanca@unica.it (M.L.M.); 2 Molecular Biology Service Laboratory, Department of Surgical Science, University of Cagliari, 09124 Cagliari, Italy; orru@unica.it (G.O.); s.fais@unica.it (S.F.); a.scanoa@unica.it (A.S.); 3 Biopharmaceutics and Pharmacokinetics Unit, Institute for Nanoscience and Nanotechnology, University of Barcelona, 08007 Barcelona, Spain; eescribano@ub.edu; 4 Department of Range and Watershed Management, Faculty of Natural Resources and Earth Sciences, University of Kashan, Kashan 8731753153, Iran; mansurehghavam@gmail.com; 5 Department of Food Science, College of Agriculture, University of Tabriz, Tabriz 5166616471, Iran

**Keywords:** quercetin, phospholipid vesicles, glycols, ethanol, oral mucosa, oral cavity bacteria

## Abstract

The aim of this work was the simultaneous loading of quercetin and mint essential oil (mint oil) in phospholipid vesicles specifically tailored to obtain an antibacterial and antioxidant mouthwash. The vesicles were prepared using soy lecithin and Tween 80 as bilayer components, and a mixture of phosphate buffer solution (33%), propylene glycol (33%) and ethanol (33%) as dispersing phase. The formation of regularly shaped, spherical and unilamellar vesicles was confirmed by cryogenic transmission electron microscopy analyses. Similarly, light scattering results disclosed that the size of the vesicles increased by increasing the concentration of mint oil, but at the same time the high amount of mint oil ensured high stability, as the size of these vesicles remained unchanged during 12 months of storage. All tested formulations were highly biocompatible towards epithelial cells and capable of counteracting oxidative cell damages caused by hydrogen peroxide. Moreover, the vesicles prepared with the highest concentration of mint oil inhibited the proliferation of the cariogenic *Streptococcus mutans* (*S. mutans*) and *Lactobacillus acidophilus* (*L. acidophilus*).

## 1. Introduction

Quercetin is one of the most abundant natural flavonoids ubiquitously found in foods including fruits, vegetables, tea and wine [1]. Flavonoids are secondary metabolites produced by plants to help their growth and development, protect them against adverse environmental constraints and provide visual and olfactory attractants for insects [2]. From the chemical point of view, flavonoids are polyphenols based on a flavan nucleus consisting of a tricyclic molecule of two phenolic rings linked to a pyranose ring [3]. Quercetin, having five hydroxylic groups placed at the 3, 3′, 4′, 5 and 7 positions, is a penta-hydroxyflavone well known for its multiple biological and beneficial effects such as antioxidant, antiaging, anti-inflammatory, prebiotic and metabolomic modulation activities. Due to this structure, it can be easily oxidized thanks to its capability of scavenging free radicals, which are involved in crucial physiological functions. Unfortunately, when over-accumulated in tissues and cells, free radicals react with membrane lipids and DNA causing lipid peroxidation and mutagenic lesions [4]. In addition, these reactive species are associated with thickening of the dermis, senescence and increased cellular permeability leading to dead cells, which in turn activate the overexpression of the proteins p16 and p21 and cause the loss of enzymatic functionality [5]. Previous studies confirmed the promising effectiveness of quercetin mainly related to the ability of scavenging such molecules. Indeed, this flavonoid can neutralize highly aggressive species such as peroxynitrite and the hydroxyl radicals, resulting in an important tool for the treatment of several diseases [4] Despite its high potential, its application in the treatment of oral diseases has not been deeply explored yet, even if it can exert not only antioxidant effects but also antibacterial activity. Different studies confirmed its ability to inhibit the grown of Gram+ and Gram− bacteria through the inactivation of extracellular proteins. Especially, it is capable of keeping the oral microflora in healthy conditions or re-establishing oral health, avoiding the onset of the most common oral diseases such as dental caries or periodontitis [6,7,8]. However, due to its low skin and mucosal penetration, its efficacy is limited, especially when administered in conventional dosage forms, but it can be improved by its incorporation into phospholipid vesicles specifically formulated for mucosal administration [9]. Due to their biphasic lamellar structure and cell affinity, these vesicles may ensure optimal performance and security, especially in the delivery of natural molecules [10]. They may improve the effectiveness of quercetin, achieving a higher deposition in the deeper skin layers thanks to their structure, which seems to be the main factor in determining the extent of the depth of deposition of quercetin in the skin [11]. Phospholipid vesicles, such as liposomes and glycerosomes, ameliorated the delivery of quercetin and its deposition in the deeper strata of the skin to a better extent than quercetin aqueous dispersion, which provided an accumulation confined to the skin surface [12]. Unfortunately, previous studies were mainly focused on the advantages connected with the loading of quercetin in phospholipid vesicles specifically designed to tackle skin damages, while no study has been published on the protection of oral mucosa using quercetin loaded in phospholipid vesicles [13,14]. Additionally, the versatility of these vesicles allows the simultaneous delivery of quercetin in association with other active molecules, to address a complementary activity and a higher effectiveness.

Considering this lack of knowledge, in the present study quercetin was co-loaded with mint essential oil (mint oil) in liposomes specifically designed for the delivery of these active molecules to the oral mucosa. Vesicles were enriched with Tween 80 and a blend of phosphate buffer and propylene glycol, and ethanol was used as hydrating medium [15]. Indeed, these water co-solvents usually improve the features and stability of liposomes [16]. Vesicles were fully characterized, evaluating their structure, morphology, size, size distribution, surface charge and stability on storage. The biocompatibility of the formulations and their ability to protect the epithelial cells from the damages induced by oxidative stress were evaluated, along with their antibacterial properties against the cariogenic bacteria *S. mutans* and *L. acidophilus*.

## 2. Materials and Methods

### 2.1. Materials

Soy lecithin (Phospholipon 90) was purchased from Lipoid GmbH (Ludwigshafen, Germany). Propylene glycol, ethanol, DPPH (2,2-diphenyl-1-picrylhydrazyl), quercetin and all other reagents and solvents of analytical grade were purchased from Sigma-Aldrich (Milan, Italy). Cell medium, fetal bovine serum, penicillin and streptomycin were provided by Life Technologies Europe (Monza, Italy).

### 2.2. Vesicle Preparation

Quercetin (15 mg/mL), mint oil (25, 50, 100, 200 mg/mL), phospholipon90 (P90, 60 mg/mL) and Tween 80 (13 mg/mL) were dispersed in a blend of phosphate buffer, propylene glycol and ethanol (1:1:1) to produce the vesicles (Table 1). The dispersions were hydrated for 24 h at 25 °C to facilitate the swelling of lecithin, and then sonicated 20 + 20 cycles (5 s ON and 2 s OFF; 13 microns of probe amplitude) with a high intensity ultrasonic disintegrator (Soniprep 150, MSE Crowley, London, UK). To allow the cooling of the sample, the two sonication cycles were interspersed with a 5 min break. The non-entrapped quercetin and mint oil were separated from the vesicle dispersions by dialysis in water (4 L). Each dispersion (1 mL) was transferred into a dialysis tube Spectra/Por^®^ (12–14 kDa MW cut-off, 3 nm pore size; Spectrum Laboratories Inc., DG Breda, The Netherlands). The purification process was carried out for 2 h at 25 °C under stirring, refreshing the medium after 1 h. The entrapment efficiency (EE%) of active molecules was calculated as the percentage of antioxidant activity found after dialysis versus that initially measured. The antioxidant activity was measured by the DPPH colorimetric test. The DPPH test has been used to measure the amount of incorporated active molecules as a function of their antioxidant activity, as it is a reliable and repeatable method, which led to the detection of both actives with a single analysis.

### 2.3. Vesicle Characterization

The formation and morphology of the vesicles were evaluated by cryogenic transmission electron microscopy (cryo-TEM). Each sample was adsorbed on a grid covered with a perforated carbon film. An automatic plunge freezing apparatus (Vitrobot, FEI Company, Eindhoven, The Netherlands) was used to vitrify the obtained film. The grid was plunged (kept at room temperature and 100% humidity) into ethane, which was maintained at its melting point using the Vitrobot. A Tecnai F20 microscope (FEI Company) was employed to observe the vitrified films at ~−173 °C and 200 kV. A CCD Eagle camera (FEI Company) was used for acquiring the digital images at low-dose imaging conditions.

The average diameter and polydispersity index, which indicate the width of size distribution, were determined by dynamic light scattering by using a Zetasizer nano-ZS (Malvern Instruments, Worcestershire, UK). The Zetasizer nano-ZS was also used for zeta potential estimation by converting the electrophoretic mobility through the Smoluchowski approximation of the Henry equation. Samples (*n* = 6) were properly diluted (1:1000) with water and analysed at 25 °C.

### 2.4. Stability Study

The formulations were stored in the dark at 25 °C and the stability was evaluated, measuring vesicle average size, polydispersity index and zeta potential monthly for 12 months.

### 2.5. Vesicle Behaviour at Salivary pH

Artificial saliva solution was prepared by dissolving 2.38 g of Na_2_HPO_4_, 0.19 g of KH_2_PO_4_, and 8 g of NaCl in 1 L of water. The pH was adjusted at 6.75 with phosphoric acid [17]. The vesicles were diluted (1:1000) with salivary medium and maintained at ~37 °C for 10 min. The mean diameter, polydispersity index and zeta potential of the vesicles were measured at the end of each incubation period [18].

### 2.6. Cell Viability and Protection against Oxidative Stress

Immortalized human keratinocytes (HaCaT) were grown (37 °C, 100% humidity and 5% CO_2_) using Dulbecco’s Modified Eagle Medium (DMEM), with high glucose supplemented with 10% (*v*/*v*) foetal bovine serum, penicillin (100 U/mL) and streptomycin (100 µg/mL), as a growth medium. The cells were seeded into 96-well plates at a density of 7.5 × 10^3^ cells/well. After 24 h of incubation, cells were treated for 48 h with vesicle formulations at different dilution (1:1000, 1:10,000, 1/100,000, 1:1,000,000). The selected incubation time seemed to be ideal to have reliable and repeatable results [19,20,21]. Cell viability was determined by adding MTT [3(4,5-dimethylthiazolyl-2)-2, 5-diphenyltetrazolium bromide] (100 µL, 0.5 mg/mL) to each well, dissolving (after 3 h) the formed formazan crystals with dimethyl sulfoxide and measuring the absorbance at 570 nm with a microplate reader (Synergy 4 Reader, BioTek Instruments, AHSI S.p.A, Bernareggio, Italy). The viability of treated cells was calculated as the percent of viable cells in comparison with that of non-treated control cells (100% viability).

The in vitro protective effect of the formulations against cell damages caused by oxidative stress was evaluated as well. The cells were seeded into 96-well plates at a density of 7.5 × 10^3^ cells/well. After 24 h of incubation, the cells were stressed with hydrogen peroxide (1:40,000 dilution) and treated with quercetin (15 mg/mL) and mint oil (25, 50, 100, 200 mg/mL) loaded in vesicles and diluted 1/10,000 with the growth medium. Cells treated with quercetin (15 mg/mL) and mint oil (200 mg/mL) dispersed in a blend of propylene glycol, ethanol and water, and diluted as the samples (1:1000), were used as control, whereas cells stressed with hydrogen peroxide and untreated were used as the negative control. After 4 h of incubation, the cells were washed with fresh medium, and their viability was determined by the MTT assay. The results are reported as the percentage of untreated cells (100% viability).

### 2.7. Determination of Antibacterial Activity

As previously reported, the antibacterial activity of the formulations was evaluated by means of the inhibition halo test using planktonic cultures of *S. mutans* (ATCC 35668) and *L. acidophilus* (ATCC 4356) [22]. The bacterial culture was performed in Mueller–Hinton Agar by adding sheep blood for all the strains and aseptically transferring the strains into 2 mL of sterile saline by a sterile loop. The turbidity of the bacterial suspensions to the required range for bioassays (1–2 × 10^8^ UFC/mL) was adjusted according to the McFarland standard as a reference. The experiment was performed by absorbing the formulations (15 μL) on blank sterile paper disks (6 mm) and placing them in the centre of Petri dishes (9 cm in diameter) of Muller–Hinton inoculated with 0.1 mL of bacterial suspension. The plates were refrigerated at 4 °C for 2 h to allow the diffusion of the sample into the agar medium and then incubated at 37 °C for 48 h. The tests were performed in triplicate, and the inhibitory activity was calculated as a function of the inhibition halo diameter. The growth of the bacteria (0% inhibition) was considered as the positive control and prepared using a paper disk without the formulations. The negative control was prepared using gentamycin (10 mg/disk; Gibco). The bioassays were performed following the protocols of the Clinical and Laboratory Standards Institute (CLSI), formerly the National Committee for Clinical Laboratory Standards, and using a biological safety cabinet.

### 2.8. Statistical Analysis of Data

Results are expressed as the means ± standard deviations. Multiple comparisons of means (ANOVA) were used to substantiate statistical differences between groups, while Student’s *t*-test was used to compare two samples. The significance level of 0.05 and the software package of XLStatistic for Microsoft Excel were used to analyse the data.

## 3. Results

### 3.1. Physico-Chemical and Technological Characterization of Vesicles

**Figure 1:**
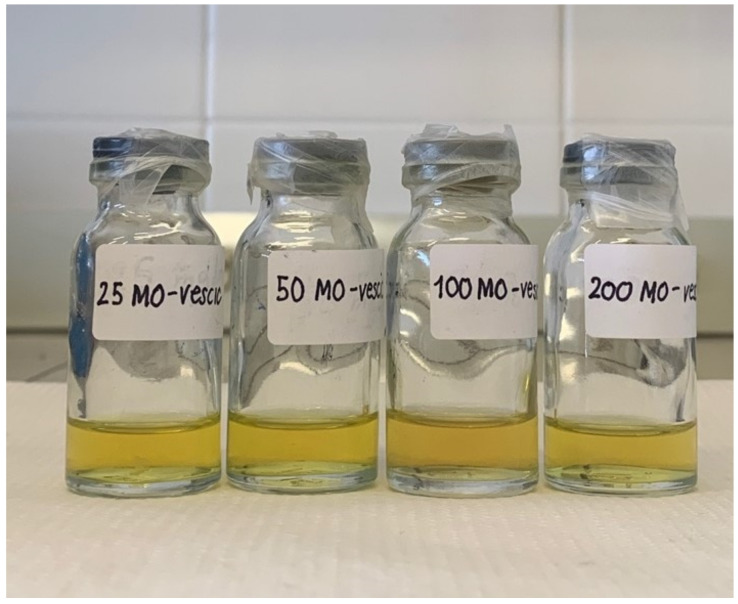
Representative images of the different dispersions of vesicles loading quercetin and increasing amounts of mint oil.

In a preliminary step, quercetin and mint oil loaded liposomes were prepared with buffered aqueous solutions at different pH; moreover, increasing concentrations of quercetin, mint oil and phospholipids were tested. The resulting vesicles were large in size (>600 nm), had a high polydispersity index and limited stability, leading to precipitation phenomena in a short time. Thus, Tween 80 was added, and the water phase was partially replaced with propylene glycol and ethanol (1:1:1 *v*/*v*). The obtained formulations were slightly yellow-coloured and transparent, with pleasant organoleptic characteristics (Figure 1) associated with an intense mint aroma, therefore suitable to be used as a mouthwash for oral hygiene.

The vesicles loading 25, 50 and 100 mg/mL of mint oil had the same average diameter, ranging from 115 ± 8 nm to 122 ± 9 nm (*p* > 0.05 among the values), the same polydispersity index ~0.28 and the same zeta potential ~−16 mV (Table 2). In addition, when the concentration of mint oil was increased up to 200 mg/mL, the corresponding vesicles had a higher mean diameter ~145 (*p* < 0.05 versus the values of other samples) and a more negative zeta potential ~−25 mV (Table 2). The entrapment efficiency was about ~100% irrespective of the formulation used, confirming the optimal delivery performances of vesicles (Table 2).

**Figure 2:**
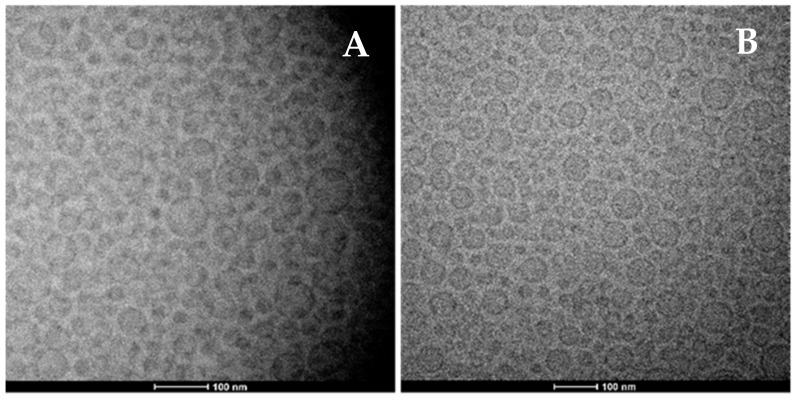
Representative cryo-TEM images of vesicles loading quercetin (15 mg/mL) and 25 mg/mL of mint oil ((**A**), left panel), or quercetin (15 mg/mL) and 200 mg/mL of mint oil ((**B**), right panel).

**Figure 3:**
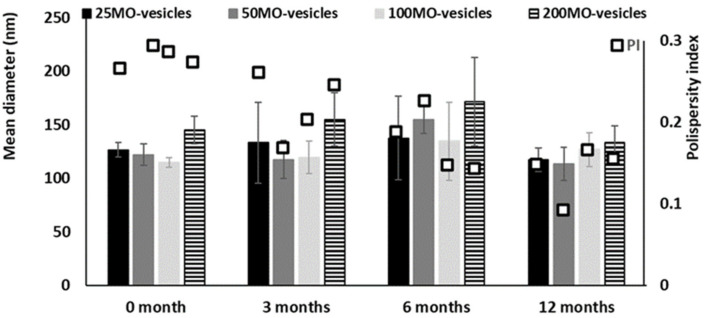
Mean diameter and polydispersity index of vesicles loading quercetin (15 mg/mL) and increasing amounts of mint oil (25, 50, 100 and 200 mg/mL) and stored for 12 months at 25 °C. Mean values (bars) ± standard deviations were reported (*n* = 3).

The mean diameter, the polydispersity index and the zeta potential of vesicles were monitored and measured at scheduled time points during 12 months of storage (Figure 2). Vesicle dispersions were stable; indeed, the mean diameter and zeta potential (data not shown) remained constant (*p* > 0.05 among the values) and polydispersity index decreased up to ~0.18 (Figure 3).

In the light of their possible use for the oral cavity, the behaviour of the vesicle dispersions after dilution and incubation with the artificial saliva medium at pH 6.75 was evaluated (Table 3). The dimensional parameters of the formulations were highly affected by the medium and underwent an increase inversely proportional to the concentration of mint oil loaded in the vesicles. Indeed, the average diameter of the vesicles loading 25 and 50 mg/mL of mint oil was 5 times larger (~ 580 nm) in comparison with that of undiluted vesicles, and the polydispersity index also increased up to ~0.49. The mean diameter of vesicles loading 100 mg/mL of mint oil increased less, ~3.5 times (~411 nm), and those of vesicles loading 200 mg/mL of oil doubled their value (~289 nm) and the polydispersity index was ~0.37, indicating a sample slightly polydispersed. The zeta potential of all formulations increased, reaching almost zero, due to the neutralization of the negative groups of phosphatidylcholine at this pH and ionic strength (Table 3).

### 3.2. Evaluation of Biological Properties of Vesicle Formulations

**Figure 4:**
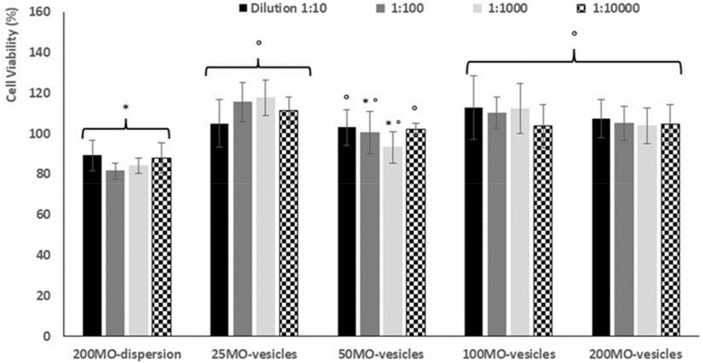
Viability of cells treated with quercetin and mint oil (200 mg/mL) in dispersion or with vesicles loading quercetin and mint oil (25, 50, 200, 200 mg/mL) properly diluted. Mean values (bars) ± standard deviations were reported (*n* = 8). The same symbol (*, °) indicates the same value.

To evaluate the possible use of the formulation in the oral cavity, biocompatibility was assessed using immortalized keratinocytes, which are the main cells of both skin surface and mucosae. Formulations were added to the culture medium of keratinocytes at different dilutions (1:10, 1:100, 1:1000, 1:10,000) and incubated for 48 h (Figure 4). Quercetin and mint oil (200 mg/mL) dispersed in a blend of phosphate buffer, and propylene glycol and ethanol, at the same dilutions, were used for comparison to estimate the carrier toxicity.

Using the dispersion, the viability of the keratinocytes was ~85%, regardless of the dilution used (*p* > 0.05 among the values obtained at different dilutions). The incubation of cells with quercetin and mint oil loaded vesicles ensured higher cell viability; however, using vesicles loading 50 mg/mL of mint oil at the intermediate dilutions (1:100, 1:1000), the cell viability was not statistically different with respect to that of cells incubated with the dispersion. The results indicated that quercetin and mint oil, at the concentrations tested, have negligible toxic effects and their loading in the phospholipid vesicles permits obtainment of safe and highly biocompatible formulations.

**Figure 5:**
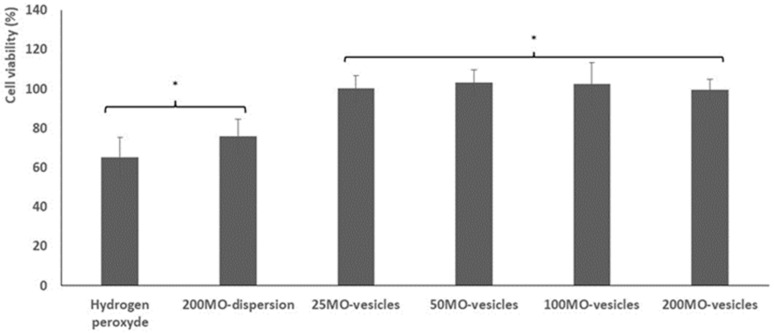
Viability of cells stressed with hydrogen peroxide and treated with the dispersion containing quercetin and mint oil (200 mg/mL) or with the vesicles loading quercetin and mint oil (25, 50, 200, 200 mg/mL) properly diluted. Mean values (bars) ± standard deviations were reported (*n* = 8). The same symbol (*) indicates the same value.

The ability of the vesicles to protect keratinocytes from oxidative stress caused by hydrogen peroxide was evaluated and compared to that of quercetin and oil (200 mg/mL) dispersed in the blend of phosphate buffer, propylene glycol and ethanol (Figure 5). 

The viability of cells stressed with hydrogen peroxide and not treated with the formulations was ~67% (*p* > 0.05 versus that cells treated with dispersion), and did not increase significantly (~75%, *p* < 0.0 versus the values obtained using vesicles) when quercetin and mint oil in dispersion were used. On the contrary, when the cells were treated with quercetin and mint oil loaded vesicles, regardless of the amount of oil used, the viability reached ~100% (*p* < 0.05 among the groups and *p* > 0.05 versus the values obtained with cells untreated and treated with dispersion), confirming the formulation’s ability to counteract the formation of peroxide radicals, thus avoiding cell damage and death. 

Considering that quercetin and, especially, essential oils are well known for their antimicrobial properties, the antibacterial efficacy of the formulations was measured as well against some pathogens capable of modifying the homeostasis of the oral cavity: *S. mutans* and *L. acidophilus* (Table 4). Only the treatment with the highest concentration of mint oil (200 mg/mL) allowed the detection of antibacterial activity, as the measured inhibition halos were ~11 mm against *S. mutans* and ~8 mm against *L. acidophilus*, confirming the suitability of this formulation for the protection of the oral cavity. The inhibition halo provided by vesicles was statistically equivalent to that provided by the dispersion, denoting that vesicles did not ensure a better efficacy.

## 4. Discussion

Quercetin and mint oil were simultaneously loaded in liposomes, aiming to prepare an antioxidant and antibacterial formulation for the treatment of the oral cavity. The liposomes initially prepared were large and precipitated quickly. Therefore, Tween 80 was added and a blend of phosphate buffer, propylene glycol and ethanol (1:1:1% *v*/*v*) was used as hydrating medium. Using these additives, small and stable vesicles were obtained. As previous reported, the combination of surfactants and water co-solvents may modify the bilayer assembling of phospholipids, increasing the incorporation of lipophilic molecules inside [23]. Indeed, in previous studies, quercetin was incorporated in high amounts (10 mg/mL) in glycerosomes or other phospholipid vesicles containing penetration enhancers [12,24]. It was the highest concentration to be loaded, using these vesicles, which contained at maximum 50% of water co-cosolvents. In the present study, the concentration of water co-solvents was improved up to 66% and was combined with the addition of Tween 80. Thanks to this combination, 15 mg/mL of quercetin was loaded and combined with increasing concentrations of mint oil, 25, 50, 100 and 200 mg/mL, which were tested to select the most suitable formulation. The vesicles were prepared in a sustainable way by direct sonication, thus minimizing the use of toxic organic solvents and additional time-consuming steps [24]. The loading of the oil at lower concentrations up to 100 mg/mL did not significantly affect the vesicle features, while when using 200 mg/mL of mint oil a change in the vesicle shape and assembly was detected, probably because the high amount of oil better intercalated inside the bilayer, modifying its curvature radius and forming larger vesicles, according to results reported for clove essential oil [25,26]. Precipitation phenomena were observed using higher concentrations of oil (>200 mg/mL) beyond 200 mg/mL, the critical value to be entrapped, probably due to an unbalanced oil and phospholipid ratio. The blend of the selected co-solvents and Tween 80 added during the formulation process allowed obtaining yellow transparent dispersions containing small, spherical, and homogeneously dispersed vesicles. Thanks to their solubilizing properties, these components affected the features of vesicles facilitating the entrapment of the two active molecules and the formation of transparent dispersions, as previously reported [17]. At the same time, Tween 80 worked as an edge activator of vesicle bilayer, making it more deformable, while water co-solvents modified the vesicle assembly and acted as skin penetration enhancers [15,27,28,29,30]. Therefore, together they exerted a synergic effect facilitating the delivery of active molecules through the mucosal barrier and inside the cells; this justified the improved effect of quercetin and mint oil loaded vesicles observed in counteracting oxidative damage in cells [31]. Thanks to the addition of these components, all the prepared formulations were stable and remained unchanged for 12 months of storage. After dilution with artificial saliva, vesicles loaded with 200 mg/mL were more stable than those containing lower concentrations of oil, denoting a higher resistance to the medium and their suitability for the treatment of the oral cavity where saliva secreted by salivary glands is present. Saliva is a slightly acidic complex fluid consisting of 90% of the liquids secreted by the salivary glands and 10% of crevicular fluid characterised by high acidity [32]. Thus, the H^+^ ions of the saliva medium neutralized the negative groups of phosphatidylcholines, breaking down the zeta potential of vesicles and favouring the bilayer enlargement. However, considering the low contact time between the mouthwash and the oral mucosa, and the expulsion of the product after rinsing the mouth, these data do not seem to negatively affect the efficacy of the nanoformulations. Formulations were biocompatible and capable of protecting the cells from the damages caused by hydrogen peroxide thanks to the ability of vesicles to interact with them, thus promoting the internalization and the synergic effect of quercetin and mint oil inside them [33,34]. The ability of mint oil to counteract oxidative stress has been previously demonstrated and is related to the different terpenes contained in the essential oil, which react with peroxyl radicals [35]. The undoubted antioxidant activity of quercetin is well known and has been confirmed by numerous studies that have been performed to gather scientific evidence for these beneficial health claims as well as data regarding the exact mechanism of action and possible toxicological aspects of this flavonoid [36]. Finally, the microbiological results suggested that the well-known antimicrobial activity of both active molecules was improved by their incorporation in vesicles, but only when the highest amount of mint oil (200 mg/mL) was used, as they were capable of inhibiting the growth of two representative pathogens of the oral cavity: *S. mutans* and *L. acidophilus*. Probably, lower concentrations of essential oil (25, 50 and 100 mg/mL) were not enough to counteract the growth of pathogens, confirming the key role of oil in antibacterial activity of formulations [27,37]. The efficacy is due to its chemical composition, which allows interference with the functionality of wall proteins that are involved in the processes of transport of compounds in the pathogen membrane [38]. 

In addition to these promising biological activities, the prepared formulations had pleasant organoleptic properties associated with an intense mint aroma, resulting in being suitable for use as a mouthwash for oral hygiene. Thus, overall results are predictive of their possible use for the protection of the oral cavity, which is the site of the access of food and sugar drinks. It is continuously exposed to many external chemical and biological agents or mechanical factors that may cause alterations in the equilibrium of its precious and complex microbiota [30,38]. The last is considered the second most complex ecosystem of the human body, after the gastrointestinal one. Thus, an appropriate care of the oral cavity provides an important protection, which in turn may improve quality of life [38]. Accordingly, in the last decades, the use of natural compounds emerged as a safe and effective alternative to synthetic antimicrobials due to the large number of strains resistant to a wide variety of chemicals [39]. The system proposed in this study is a natural and advanced nanoformulation which fully responds to these needs. 

## 5. Conclusions

Overall results confirmed the promising properties of quercetin and mint oil liposomes improved with Tween 80, propylene glycol and ethanol. Indeed, they form a yellow transparent dispersion with a pleasant aroma of mint, with small vesicles highly stable in storage. The tailored systems were highly biocompatible and capable of counteracting oxidative and bacterial damages. Considering these findings, the vesicle dispersion loading 15 mg/mL of quercetin and 200 mg/mL of mint essential oil can be proposed as effective antibacterial and antioxidant mouthwash for the protection of the oral cavity. 

## Figures and Tables

**Table 1:** Composition of quercetin and mint oil-loaded vesicles prepared with phospholipon 90 (P90) and Tween 80, and hydrated with a mixture of phosphate buffer, propylene glycol and ethanol (1:1:1 *v*/*v*).

	P90 (mg/mL)	Quercetin (mg/mL)	Tween80 (mg/mL)	Mint oil (mg/mL)
25MO-vesicles	240	15	13	25
50MO-vesicles	240	15	13	50
100MO-vesicles	240	15	13	100
200MO-vesicles	240	15	13	200

**Table 2:** Mean diameter (MD), polydispersity index (PI), zeta potential (ZP) and entrapment efficiency (EE) of quercetin and mint oil loaded vesicles. Each value represents the average ± standard deviation of at least six replicates.

	MD (nm)	PI	ZP (mV)	EE (%)
25MO-vesicles	* 122 ± 9	0.28	−16 ± 3	99 ± 2
50MO-vesicles	* 119 ± 13	0.29	−17 ± 3	100 ± 2
100MO-vesicles	* 115 ± 8	0.29	−15 ± 4	99 ± 1
200MO-vesicles	° 145 ± 14	0.29	−25 ± 3	100 ± 1

The morphology and structure of the vesicles observed using the cryo-TEM confirmed the formation of spherical and unilamellar vesicles, regardless of the amount of mint oil used (Figure 2). *, ° indicate the same values.

**Table 3:** Mean diameter (MD), polydispersity index (PI) and zeta potential (ZP) of vesicles diluted with simulated saliva medium at pH 6.75. Diluted samples were stored at 37 °C for 10 min. Mean values (*n* = 6) are reported ± standard deviation.

	MD (nm)	PI	ZP (mV)
25MO-vesicles	579 ± 14	0.49	−1± 2
50MO-vesicles	590 ± 28	0.48	−1 ± 3
100MO-vesicles	411 ± 46	0.42	−1 ± 1
200MO-vesicles	289 ± 4	0.37	−1 ± 2

**Table 4:** Inhibition halo (IH) of quercetin and peppermint oil (200 mg/mL) in dispersion and vesicles loaded quercetin and mint oil (200 mg/mL) against *S. mutans* and *L. acidophilus*. Mean values ± standard deviations are reported.

Sample	*S. mutans* IH (mm)	*L. acidophilus* IH (mm)
200MO-dispersion	10 ± 4	8 ± 3
200MO-vesicles	12 ± 3	9 ± 3

## Data Availability

The data is contained within the article.
